# Dr. R. W. Scott, on Bronchorrhœa Æstiva, or Hay-Fever

**Published:** 1842-07-02

**Authors:** R. Wakefield Scott

**Affiliations:** Liverpool


### ANALECTA.

*On Bronchorrhœa Æstiva, or Hay-Fever.* By R. Wakefield Scott, M. D., Liverpool.—As the season is approaching at which this peculiar affection occurs, and as your Journal possesses an extensive circulation in the rural districts, I am induced to offer a few observations respecting its pathology, and the means which I have found effectual in shortening its duration, and preventing its recurrence.

I have had an opportunity during the last few years of observing the origin and progress of a very obstinate case of this disease, which had recurred annually for fifteen years, usually continuing during the entire summer, and ceasing only at the commencement of autumn, and confining the patient almost wholly to the house.

It had been previously considered as an ordinary catarrh, occurring at an unusual season, and I must confess that it was not for some time that it appeared to me identical with the complaint known as the hay-fever or asthma, of which, though aware of its existence, I had seen no previous example.

The case was that of an unmarried lady, of middle age, of lax fibre, and leucophlegmatic temperament, in other respects enjoying good health, but who had, until the summer of last year, suffered attacks of greater or less severity during the period which I have mentioned.

The disease commenced with the symptoms of a common catarrh—sneezing, itching, and running of the eyes and nose, great dyspnœa, with a distressing cough, continuing occasionally, with little intermission for two or three hours, and accompanied with a profuse expectoration of thin frothy mucus, great languor and debility, with a feeling of weight and pain in the spine at the dorsal and lumbar regions, a quick and weak pulse, but without fever. She had been treated without the slightest benefit by leeches and blisters, expectorants and antispasmodics, and protracted abstinence. Upon inquiry into the history of the disease, I ascertained,—

1. That the time of its recurrence was irregular—that it took place very early in the summer, if the temperature was unusually high, and *vice versa* —that on the first or second very warm day, especially if suddenly supervening, at whatever period this change occurred, the sneezing, languor, and dyspnœa commenced—that the severity of the symptoms bore a relation to the elevation of temperature—and that its recurrence was retarded by removal to a colder situation.

2. I found, also, that exposure to cold air afforded relief.

3. That the symptoms were aggravated by a debilitating regimen and antiphlogistic treatment.

4. That an occasional stimulant, as wine and porter, afforded much relief.

5. And that expectorants and antispasmodics were useless, and purgatives hurtful.

From all these circumstances it seemed clear that the disease was one of mere relaxation and passive congestion, especially at the mucous membrane of the air passages and of the lungs, exciting the dyspnœa and cough, and which were temporarily relieved by increased secretion.

The diminution of atmospheric pressure, the expansion of the blood, and the relaxation of the solids produced by increased temperature, especially if occurring rapidly, may, undoubtedly, in certain constitutions, lead to congestion of particular organs, by subverting the balance which must exist between the quantity and momentum of the blood and the resisting power of their capillaries.

The influence of this increase of temperature being general, its effects ought to be more or less general also, and hence, I apprehend, it will be found that most individuals of delicate constitutions and lax fibres, whose vessels are less capable of resisting any sudden change in the circulation, are subject to congestion, more or less general, at these particular seasons ; in most cases the congestion is general, and occasions only a state of languor and oppression, and is relieved by increased perspiration ; but in others it occurs locally, giving rise to diarrhœa, augmented biliary or urinary secretion, or hæmorrhage ; and it is, I conceive, some comparative debility or minor contractile and resisting power, in the vessels of certain parts, as compared with those of others, which determines the locality of the congestion in particular individuals ; and that it is this comparative debility of the capillaries of the air-passages and the lungs, aided perhaps by increased nervous irritability, which gives the predisposition, which must necessarily exist for the production of this species of bronchorrhœa.

Conceiving, then, that a general loss of tone, especially in the parts affected, was the origin of the complaint, it was obvious that the most appropriate mode of treatment must consist in strengthening the constitution generally, in which the vascular system might so far participate as to be able to maintain the equilibrium of the circulation during that change in its condition.

The plan which I adopted was to recommend a more nutritious diet, consisting of more animal and less vegetable food, and less fluids than ordinary, with one or two glasses of port-wine at dinner ; the use of the shower-bath, which was persevered in during one entire year ; and subsequently cold sponging of the throat, chest, and back, with an occasional aperient; and from one to three grains of the sulph. quinæ in infus. cuspariæ three times a-day.

This plan was commenced about a fortnight before the expected attack, and had the effect, in the first summer that it was adopted, of considerably delaying its recurrence, of lessening its severity, and shortening its duration. This was the case, in a still more marked degree, in the subsequent summer, the attack only continuing for a few days during the greatest heat, and during the whole of last summer the complaint was entirely absent. This tonic plan of treatment, which I find was recommended by Mr. Gordon some time ago, was continued during the existence of the disease with decided benefit, as it became much milder, and of shorter duration; but I consider the most important object to be, so to prepare the system beforehand as to prevent its recurrence.

This affection, as the ordinary term applies, has been usually, and is by many still considered to arise from the aroma of hay, or other vegetable substances, but, as I conceive, on no substantial grounds. In the case which I have related the attacks frequently occurred long before any grass had been cut, and in situations to which no such effluvia could be supposed to penetrate; the apparent connection has evidently arisen from the same degree of temperature which causes the grass to ripen, producing peculiar effects in certain constitutions. There is no doubt that the aroma of hay will readily produce a paroxysm in those affected, or may hasten the attack in the predisposed; but these are effects which it possesses only in common with other irritations, as the perfume of the bean or potato-flower, or dust, indigestible food, and mental emotions.

The non-occurrence of the usual attack, or its minor severity when the individual removes to the sea-coast, which has been supposed to depend upon the absence of vegetable effluvia, are much more satisfactorily accounted for by the lower temperature, the refreshing breezes, and the increased tone of the general system produced by such a locality. Various other circumstances which might at first appear to favour the idea of its connection with such emanations, may, I conceive, be readily explained in accordance with the above views.—*Prov. Med. Journ.* May 21, 1842.

